# Name and Describe the Causes of Dental Caries and the Vital, Structural, and Chemical Changes Occurring during This Process

**Published:** 1889-05

**Authors:** H. H. Schumann

**Affiliations:** W. of M.


### COMMUNICATIONS.

### Name and Describe the Causes of Dental Caries, and the Vital, Structural, and Chemical Changes Occurring During this Process.

BY H. H. SCHUMANN,

W. OF M.

Caries is a very destructive process on an important class of organs, attacking the hard structures in the human economy as gangrene does the soft tissues, *i. e.*, particle by particle. It destroys the integrity of the teeth of children as well as those of adults. The changes caries produces in the teeth are threefold, viz.: structural, chemical, and physical. When I say that it attacks the teeth of the old as well as those of the young, I mean it may attack those of the old as well as the young. We all know that decay attacks the teeth to a far greater extent, as a general rule, before the age of puberty. This destructive process may start as soon as the teeth are erupted, and as it is sometimes seen, especially in newly erupted third molars, may even begin before the tooth has really put in its appearance.

Caries is not alike in rapidity in the teeth of old people as in those of the younger ones; it being far more destructive and rapid in the latter; this is probably due to the large amount of organic substance present in young teeth, and also to their less dense structure. This disease is usually fatal to the teeth if not timely interfered with.

The causes of caries are manifold, and may be hereditary, or non-hereditary. That there is a hereditary tendency in the teeth, to decay is well known. There are many hereditary structural defects found in the teeth ; in the enamel, as well as in the dentine, that may be the occasion of decay. Variations are found frequently in both of these structures. Again, we often find *defective fissures* in molars mostly, but sometimes also in teeth situated more anteriorly in the oral cavity, fissures whose edges are not united, that is, they may be in contact with each other, but not fused together. In examination such defective fissures are often, by careless operators, thought cavities, and are unnecessarily cut out and filled. After operations of this kind the tooth is generally more liable to decay than if left with the slightly malformed fissure.

Decay agents (as they may be termed) sometimes get access through these fissures and dissolve out the dentine, leaving but a very small opening in the enamel; even the pulps may become exposed and yet the enamel stands, showing sometimes nothing but a very minute opening in a fissure, some admitting only the points of the finest explorers, and those only with difficultly. Such structural defects are comparable with a brick wall which may be defective on account of an improper arrangement in the laying of the stones, or the bricks themselves may be poor, or the cement uniting them may be defective. The chemical composition of the dentine or enamel may be poor.

At other times we find fissures with overhanging enamel, and thus easily retaining foreign putrifying substances. Other defects are fine *tracts and spots* on the teeth which are sometimes visible to the naked eye. Again, the enamel may be *shortened* where it should come in contact with the cementum at the neck of the tooth, and it thereby leaves the dentine bare. Also in the dentine frequently structural defects are observed, as for instance in the *granular layer*, which varies considerably, sometimes being quite thick and spongy, and at other times quite thin. In a very well-developed tooth the granular layer is thin and dense and the *dentinal tubules* radiate from a common center and do not, as is often the case, *scatter* in all directions, also being *partially uncalcified.* It sometimes is seen in a tooth section that the dentinal *tubules blend* and so form *open tracts*. It is true, this defect is not very frequent, but when it is found it usually runs into the enamel also. The most frequent defect in dentine is a general softness either due to abnormal size of the dentinal tubules or to an increased amount of organic matter.

It is, as already said, well known that there is a hereditary tendency for some teeth to decay. The reason though must not necessarily be a defective structure of the tooth, but may be due to hereditary diseases.

The cause of some childrens teeth being very rapidly disintegrated by caries, just like those of either of its parents, may be the result of a similarity of action of the parts concerned in the formation of the teeth. The child's teeth are in shape and structure like those of its father or mother, whom it most resembles in all respects, and from whom it may have inherited such a diathesis (according to Harris).

If a child's teeth and the surrounding tissues are like those of either of its parents, it is not at all surprising to find them decay in like manner when brought in contact with similar irritants, and as it often occurs at the same period of life.

Among the hereditary predisposing causes of dental caries, it may be well to mention *accidental causes*; in the first place; accidental injury to the teeth happen quite frequently, and more so since foot and base ball are being so highly commended. By breaking a tooth the dentine or even the pulp may be laid bare, both being usually attacked by external irritants.

*Excessive thermal changes* are more deleterious in their results upon the teeth as it is thought, and more attention should be paid to such violence. If heat and cold rapidly follow each other it will cause a splitting or cracking of the enamel and thus give access to decay producing agents,and then retain them there.

*Abuse* and *direct violence* hardly need separate consideration; they only being briefly mentioned as predisposing causes.

*Artificial* and *defective* modes of *living* need to be discussed here and far more than I will have time to do. You all know how the food of which we partake is mechanically and chemically subdivided and is then atom by atom deposited in the different structures of the body after being brought there by the blood-corpuscles. The corpuscles then are the distributors of the nutrient material in the system both to hard and soft tissues, thus sustaining the life of the old and at the same time assisting in the production of new tissues. If now, corpuscles going to a certain part of the body are partially robbed of their load of nutrient material, or if they are in the first place not laden to the sufficient degree, nor with the pure material, then certainly parts, or the whole body will have to suffer from such a disordered condition. Dr. John Allen has estimated that every child uses half a barrel of flour every year; and it is known that there are from each barrel forty pounds of bone-forming material thrown out. Now the child of civilized nations partakes not of the wheat roughly crushed, but of finely ground flour and thereby is deprived of twenty pounds in a year of the mineral constituents which should be taken in the system in order to make hard and flinty bones and teeth. By estimating it will found that the children of our nation then have been deprived of four hundred pounds each of bone-forming material by the time they reach the age of twenty years. Then instead of having hard and flinty teeth they have teeth that are more or less destroyed by dental caries resulting from an incorrect mode of living. In comparison with people of uncultivated and older nations our adults have the most poorly organized teeth.

As another cause of dental caries let me add here a few words about *uncleanliness.* How many patients do we have from whose teeth we could not remove more or less malodorous and soft decaying food, mixed with mucus, from the upper molars? There are few who are entirely free from such deposits. These putrefying substances undoubted add greatly to the rapidity with which such teeth decay; it does not essentially benefit the odor of the patient's breath, nor his digestion.

*Disease* and *drugs* themselves and also their *reflex action* sometimes affect the teeth most remarkably.

Any increased action in one part of the system is invariably followed by diminished action in another. Thus, as Prof. Garretson says, large healing wounds produce different disturbances all over the body, as constipation sometimes produces gastritis. Supposing the body becomes diseased at an early period of life, say before the time of eruption of any of the teeth, the duties and functions of the different assimilating organs will either be checked or greatly interfered with. Certainly then the teeth as well as any other part of the body would have to suffer from such malnutrition, being deprived of a proper supply of mineral substances they will be defective in structure when erupted, and then easily give away when once attacked by caries. Any other part of the body being thus deprived of a sufficient amount of nourishment may be defective but as a general rule will be repaired by nature later; this is not so with the teeth; when once affected and their structure is in any way impaired, *they* remain defective and weak. It may be well to add here that this frequently accounts for teeth decaying in pairs, being formed and malnourished both at the same period and both being exposed to the same irritants at the same time.

During sickness the teeth are quite frequently most shamefully neglected and the oral secretions are allowed to be vitiated with impunity. Many diseases show their bad effects upon the teeth through a poorly nourished body.

Undoubtedly drugs have great influence upon the teeth, but their action is not generally as deleterious as it is thought by many. Acids taken internally are generally so well diluted and flow so rapidly by the teeth, sometimes hardly coming in contact with them, that their injury upon them is very small.

Mercurials greatly injure the teeth if taken for a long while or in large doses, but not so as to produce decay; showing its bad effects mostly on the periosteum and alveolar processes, thereby loosening them and the teeth drop out of their sockets. In placing arsenic in some teeth it happens sometimes to careless operators that some of the drug gains access to the tissue surrounding. Such a tooth will generally also loosen and drop out, the results of the drug though on the surrounding tissue may be far more destructive than those of mercurials or iodides.

In looking up this subject in the works of different authors I found such a variety in opinions and theories that I thought it might be well to try a few experiments myself in regard to the action of certain drugs upon the teeth. Allow me to add here the results I found upon three teeth.

One tooth was placed in a thoroughly sterilized test-tube filled with saliva 1 part, distilled water 1 part, and aromatic sulphuric acid 120, thus forming a mixture about the same as it would be found in the mouth of any body taking a 10 m. dose of said drug before swallowing. This with the tooth was heated up to 98½° F. and left at that temperature for six days.

*Result:* Enamel had become so soft that the surface could readily be scraped off with the fingernail.

The second tooth was with the same precautions placed in a test-tube filled with 5 parts saliva, 8 parts distilled water, and 1–30 of a grain of bichloride of mercury, thus being comparable with the fluids of the mouth after a 1–30 grain dose of corrosive sublimate. Also this was left six days under the same precautions.

*Result:* There was no change perceptible to the naked eye.

The third tooth was placed in a test-tube: 1 part saliva, 2 parts distilled water, and 1–200 part of chloride of iron, being comparable with the fluids in the mouth after a 6 m. dose of tinc. ferri. chloridi. Also this was maintained at bloodheat for six days.

*Result:* The same. That is no change.

None of the teeth were out of the mouth four seconds before being dropped into into these sterilized test-tubes filled with above fluids already at bloodheat.

I think that the above three drugs are thought the most injurious ones to the teeth. These experiments lasted six days whilst taking a dose of any of the drugs the teeth are really not in direct contact with them for more than four or five seconds. I think we can safely say that the influence of drugs is, if at all, very slight in its deleterious action upon the teeth. It is a good plan though, after partaking of any drug, to thoroughly cleanse the mouth and teeth.

The causes of dental caries may be either predisposing or exciting. As predisposing causes I have already mentioned defective constitution, either innate in the child, or as derived from either of its parents, or acquired from accidental influence to which the child has been exposed. A disordered constitution of the digestive system and lymphatic system may show its destructive effects on the teeth as well as in any other part of the body. Other predisposing causes may lie in the climate in which the individual lives; marshy lands where a great amount of malaria is found, for instance, is a poor one for the teeth. A dry and sunny country, as for instance Texas, is far preferable. Again, the period of gestation affects the teeth sometimes quite remarkably. Pregnancy oftentimes leaves badly decayed teeth in the mouth of the mother.

The existing or immediate cause of dental caries is mostly the chemical action of some acid produced by vitiated secretions of the mouth or stomach, and by decomposition of animal and vegetable substances. There are quite a number of different theories as to the exciting causes of decay.

French authorities, as Auzile, Bardette, and Fouchard are of the belief that dental caries is mostly due to the action of chemical agents.

Dr. S. K. Mitchell proved in 1796 that there was an acid present in the mouth capable of destroying the tooth-structure. Dr. W. D. Miller has proven that there is continually an acid in the mouth at times more marked than at others, more generally in the morning. This acid is formed by the chemical action of saliva on saccharine food substances, of which more or less are always retained in the mouth. I found that the enamel of a tooth after laying for forty-eight hours in pure acetic acid was so softened that it easily could be removed with the fingernail.

" Prof. Wescott has proved that malic acid, or acid of apples in the concentrated form, will rapidly attack the enamel. This accounts for teeth "standing on edge," as some say, after biting into a sour apple.

After reading Dr. Miller's work on "The Causes of Dental Caries," I think that his opinion is, that acetic acid properly would have to be introduced into the mouth to produce decay, or it may be produced by the generation of other weaker acids formed by the fermentation of saccharine particles of food in the mouth.

Decay in the teeth varies in colors from the white to the black. This may be due to substances taken into the mouth which discolors the decay but which have nothing to do with the process of disintegration itself. Or some decaying agent may after acting for some time be replaced by another, and thereby the color of the decay may be changed. Sometimes these decay agents, whatever they may be, vary in concentration at different periods; sometimes they are differently mixed. This may account for the difference in the rapidity of the decay in some mouths at different periods. The difference in the kind of acids producing decay has more to do with its color than any thing else.

Thermal changes also may have some influence upon the rapidity and quality of decay. It is a well-known fact that all corroding agents act more vigorously when in a nascent state; this also accounts for the difference in rapidity as well as color. Some are of the opinion that galvanic action is the cause of decay. This, if so, is certainly indirectly so. The galvanic current in the body does not directly decompose the teeth, but substances, setting free gases, which after combining form acids, corroding and dissolving the teeth. Most theories come to the final conclusion that decay is due to some solvent.

Dr. Abbott is of the opinion that dental caries is due to inflammatory action the same as caries of the bone, or gangrene of the soft structures. Others think that *germs* may be the cause. If decay were due to parasites eating up the tooth-structure, the decayed walls of the teeth would be dry and chalk-like, which they are not. It may be that the acid secretions of these germs dissolve the tooth-structure, so that also this theory comes like most of the others to the conclusion that decay is due to some solvent. Dr. Black's germ theory, I think, also comes to this conclusion. Such agents dissolving parts of the teeth leave them sometimes so changed physically, as to make them absolutely useless as far as use in mastication is concerned. Considering the general use of the teeth in aiding digestion we may safely say that such teeth are absolutely detrimental; vitiating the fluids in the oral cavity, also producing a malodorous breath by retaining putrifying food substances. Such a decayed tooth also adds but little to the beauty of a denture. As to structural changes decay agents do not only attack a tooth as far as some think, that is the decayed part, but much further. The next lying enamel and dentine is always more or less disintegrated and is easily broken down. Sometimes decay attacks the teeth far beyond its origin and quite frequently affects the periosteum so greatly as to cause the teeth to become loose and sometimes to drop out.

